# CRISPR/Cas9-mediated gene knockout of NANOG and NANOGP8 decreases the malignant potential of prostate cancer cells

**DOI:** 10.18632/oncotarget.4293

**Published:** 2015-06-15

**Authors:** Norihiko Kawamura, Keisuke Nimura, Hiromichi Nagano, Sohei Yamaguchi, Norio Nonomura, Yasufumi Kaneda

**Affiliations:** ^1^ Division of Gene Therapy Science, Osaka University Graduate School of Medicine, Suita, Osaka 565-0871, Japan; ^2^ Department of Urology, Osaka University Graduate School of Medicine, Suita, Osaka 565-0871, Japan

**Keywords:** NANOG, NANOGP8, gene knockout, CRISPR/Cas9, prostate cancer

## Abstract

NANOG expression in prostate cancer is highly correlated with cancer stem cell characteristics and resistance to androgen deprivation. However, it is not clear whether NANOG or its pseudogenes contribute to the malignant potential of cancer. We established *NANOG*- and *NANOGP8*-knockout DU145 prostate cancer cell lines using the CRISPR/Cas9 system. Knockouts of *NANOG* and *NANOGP8* significantly attenuated malignant potential, including sphere formation, anchorage-independent growth, migration capability, and drug resistance, compared to parental DU145 cells. *NANOG* and *NANOGP8* knockout did not inhibit *in vitro* cell proliferation, but *in vivo* tumorigenic potential decreased significantly. These phenotypes were recovered in *NANOG*- and *NANOGP8*-rescued cell lines. These results indicate that NANOG and NANOGP8 proteins are expressed in prostate cancer cell lines, and *NANOG* and *NANOGP8* equally contribute to the high malignant potential of prostate cancer.

## INTRODUCTION

Tumors include a small population of cells with stem cell-like properties, including self-renewal and tumor-initiation capacities [1–5]. These cells are often termed cancer stem-like cells (CSCs) and express pluripotency-related genes [6–9], such as NANOG, OCT3/4, and SOX2, which are essential transcription factors in embryonic stem cells (ESCs) [10, 11]. These transcription factors are involved in various somatic cancers and drive tumor development [12–18]. NANOG is expressed in various cancers, such as ovarian cancer [19], breast cancer [20], colorectal cancer [21], and prostate cancer [22], and it is enriched in CSCs [16]. CD44^+^CD133^+^ and CD133^+^ cells are markers of prostate CSCs in human prostate cancer tissues [23], and these immune cells express higher NANOG mRNA levels compared to their corresponding negative cells. Increased NANOG expression is associated with poor prognosis in various cancers, such as lung cancer [24], oral cancer [25], brain cancer [26], ovarian cancer [19], breast cancer [27], and prostate cancer [22]. Additionally, NANOG expression is negatively correlated with postoperative survival in patients with lung and ovarian cancer [19, 24], and increased NANOG expression in human prostate cancer tissues is correlated with an increased Gleason score, which is an indicator of poor prognosis [22, 28].

*NANOG* (hereinafter *NANOG1* to avoid confusion) is particularly interesting because this gene has at least 10 pseudogenes, but the sequence similarities among these genes confounds analyses of NANOG expression [29]. The *NANOGP8* pseudogene has attracted attention because only *NANOGP8* encodes the full-length NANOG1 protein with a 2-amino acid substitution, and NANOGP8 is expressed in cancer cells and increases the clonogenicity and tumorigenicity [18, 21, 22, 30] [31–34]. NANOGP8 overexpression *in vitro* promotes sphere formation and migration in a prostate cancer cell line and drug resistance in a breast cancer cell line [30]. In addition, NANOGP8-overexpressing cells form larger tumors *in vivo* in immunodeficient mice [30, 33]. However, no antibodies can distinguish NANOG1 and NANOGP8 proteins because of the high similarity between these two proteins. Therefore, the expression of NANOG1 and its pseudogenes has only been analyzed using reverse transcription polymerase chain reaction (RT-PCR) and cDNA sequencing analysis [35]. Most somatic cancer cell lines predominantly express protein-coding *NANOGP8* and non-coding *NANOGP5* with markedly less *NANOG1* expression. In contrast, human ESCs and the NTERA2 cell line, which is derived from a human teratocarcinoma, express large amounts of *NANOG1* [35]. Therefore, *NANOGP8* is likely a primary contributor of NANOG protein expression in various somatic cancers [35], including prostate cancer. However, the proportion of NANOG protein expression that comes from *NANOG1* and *NANOG8* in cancer cells is not known. The overexpression of *NANOGP8* in prostate cancer cell lines has been shown to increase migration and tumorigenic potential [30], and the overexpression of *NANOG1* has been shown to increase migration in an ovarian cancer cell line [19] and increase migration, metastasis, and tumorigenic potential in a breast cancer cell line [27]. However, these previous gain-of-function studies did not include loss-of-function analyses of NANOG1 and NANOGP8 because the sequence similarity makes individual gene knockout without off-target effects difficult. Therefore, a causal role of *NANOG1* and *NANOGP8* in cancer cells is not clear.

This study established *NANOG1*- and *NANOGP8*-knockout, DU145 prostate cancer cell lines using CRISPR/Cas9 system-mediated genetic engineering [36, 37]. In the DU145 prostate cancer cell line with endogenous NANOG1 and NANOGP8 proteins, both *NANOG1* and *NANOGP8* contributed equally to many properties associated with malignant potential in prostate cancer, including sphere formation, migration, drug resistance, and tumorigenic potential. Our findings suggest that the malignant potential of cancer cells is increased by NANOG protein expression from both *NANOGP8* and *NANOG1*.

## RESULTS

### Establishment of *NANOG1*- and *NANOGP8*-knockout DU145 cell lines and rescue cell lines

**Figure 1:**
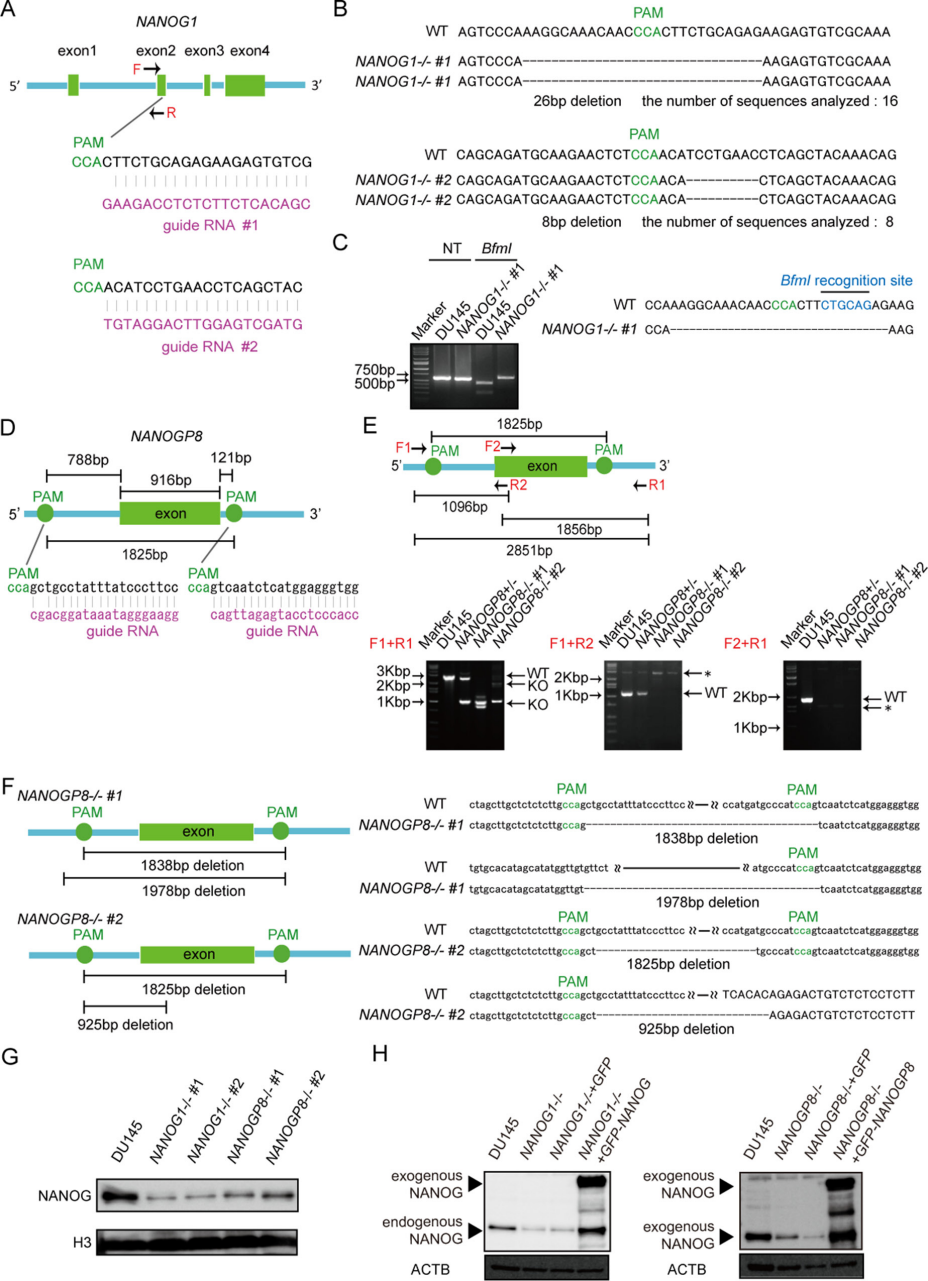
Generation of *NANOG1*- and *NANOGP8*-knockout DU145 cell lines using the CRISPR/Cas9 system **A.** Schematic representation of the *NANOG1*-targeting gRNA sequences. Arrows indicate primer positions. PAM, protospacer adjacent motif. **B.** Two *NANOG1*−/− cell lines were established from DU145 cells. The deleted sequences in the *NANOG1*−/− #1 and *NANOG1*−/− #2 cell lines are presented. The number of sequences analyzed for each cell line is indicated below the deleted sequences. **C.** Confirmation of the genotype of *NANOG1-/*- cells. The *NANOG1* genomic regions in the indicated cells were amplified using the *NANOG1*-specific primers indicated in Figure 1A. Amplicons digested by *Bfm*I were separated in the depicted agarose gel. NT, no treatment. **D.** Schematic representation of the *NANOGP8*-targeting gRNA sequences. **E.** Upper panel: Schematic representation of the *NANOGP8* genomic region, targeted PAM positions, and primer positions. Arrows indicate primer positions. Lower panel: Genotyping of *NANOGP8-/*- cells. The *NANOGP8* genomic region was analyzed by PCR. Amplicons were separated in agarose gels. Using the F1 + R1 primer set, the 2851 bp wild type region (WT) was amplified in DU145 cells, whereas shorter amplicons (KO) were detected in *NANOGP8*-knockout cell lines. Neither the F1 + R2 primer set nor the F2 + R1 primer set amplified the *NANOGP8* genomic region in *NANOGP8*−/− cells. Asterisks indicate non-specific bands. **F.** Establishment of two *NANOGP8*−/− cell lines from DU145 cells. *NANOGP8-/*- #1 cells have deletions of 1838 bp and 1978 bp, and *NANOGP8-/*- #2 cells have deletions of 1825 bp and 925 bp. **G.** NANOG protein levels in the indicated cell lines. NANOG protein expression was analyzed by Western blot analysis. H3 was used as the loading control. **H.** Establishment of *NANOG1*- and *NANOGP8*-rescued cell lines. NANOG expression in these rescued cell lines was analyzed by Western blot. Exogenous NANOG indicates GFP-NANOG and GFP-NANOGP8 proteins in each rescued cell lines. ACTB was used as the loading control.

Human *NANOG1* has at least 10 pseudogenes. *NANOG1* and the pseudogene *NANOGP8* code for intact NANOG protein. We first generated each gene knockout in DU145 cells (human prostate cancer cell line) using the CRISPR/Cas9 system to evaluate the functions of these two genes [36, 37]. We designed two gRNAs against exon 2 of *NANOG1*, which codes for the homeodomain, to avoid non-specific effects of the CRISPR/Cas9 system (Figure 1A). Genomic DNA PCR products from each cell were cloned into a plasmid to analyze the targeted *NANOG1* genomic region in each transfected cell line. The *NANOG1* PCR primers only amplify the *NANOG1* genomic region because the forward primer recognizes intron 1 of *NANOG1*, which is unique among *NANOG1* and its pseudogenes (Figure 1A). This primer amplified the targeted *NANOG1* genomic region, and amplicon sequence analyses demonstrated that *NANOG1*−/− #1 and *NANOG1*−/− #2 harbored 26 bp and 8 bp deletions, respectively, in exon 2 of the *NANOG1* gene (Figure 1B). All 16 analyzed sequences from *NANOG1*−/− #1 and all 8 analyzed sequences from *NANOG1*−/− #2 exhibited the same deletions. The amplified genomic region in DU145 cells includes a restriction enzyme cleavage site for *Bfm*I, and this cleavage site is lost in *NANOG1*−/− #1. We performed restriction enzyme analysis of the PCR amplicon to confirm the deletion in *NANOG1*−/− #1 cells (Figure 1C). *Bfm*I completely digested the amplicon from DU145 cells, but the amplicon from *NANOG1*−/− #1 cells was not digested. Therefore, we confirmed the deletion of the *NANOG1* gene on both alleles in *NANOG1*−/− #1 cells. This method to confirm the deletion was not available in *NANOG1*−/− #2 cells because the deleted site did not contain a restriction enzyme site. Therefore, we confirmed the deletion of the *NANOG1* gene on both alleles in *NANOG1*−/− #2 cells using sequencing analysis. The deletions in *NANOG1*−/− #1 and #2 cells induce frame shifts, which result in premature stop codons. We examined the off-target effects of the gRNAs on NANOG pseudogenes because the gRNA expression constructs that targeted exon 2 of *NANOG1* exhibit a high similarity to NANOG pseudogenes. In conclusion, *NANOG1*−/− #1 cells have a 19 bp and a 5 bp deletion in *NANOGP7* and a 124 bp insertion in *NANOGP9*. However, *NANOG1*−/− #2 cells harbor no indels in NANOG pseudogenes. *NANOG1*−/− cell lines exhibited no off-target effects in *NANOGP8*, which is the only NANOG pseudogene with the ability to produce intact full-length NANOG protein.

To delete the *NANOGP8* gene, we designed two gRNAs outside of *NANOGP8* (Figure 1D). Because most *NANOG* pseudogenes, including *NANOGP8*, are intronless genes with highly conserved sequences, it is difficult to specifically amplify the exon of *NANOGP8*. It is also challenging to quickly examine the genotypes of *NANOGP8*-targeted clones using the single-gRNA approach employed for *NANOG1* (Figure 1A and 1D). We designed three primer sets to screen for *NANOGP8* gene deletion. Primer set F1 + R1 amplified a 2851-bp region of the *NANOGP8* gene in DU145 cells, and the amplicon was apparently shorter in the *NANOGP8* gene knockout cell line (Figure 1E). Primer sets F1 + R2 and F2 + R1 could not amplify the *NANOGP8* genomic region in the *NANOGP8* gene knockout cell line (Figure 1E). These primers identified two *NANOGP8*−/− cell lines. Sequence analysis of the PCR products revealed that *NANOGP8*−/− #1 had a 1838-bp and a 1978-bp deletion, and *NANOGP8*−/− #2 had a 1825-bp and a 925-bp deletion (Figure 1F), which indicated that we generated two independent *NANOGP8*-knockout DU145 cell lines. Unfortunately, we were unsuccessful in our attempt to establish *NANOG1*- and *NANOGP8*-double knockout cell lines (Supplementary Figure 1).

**Figure 2:**
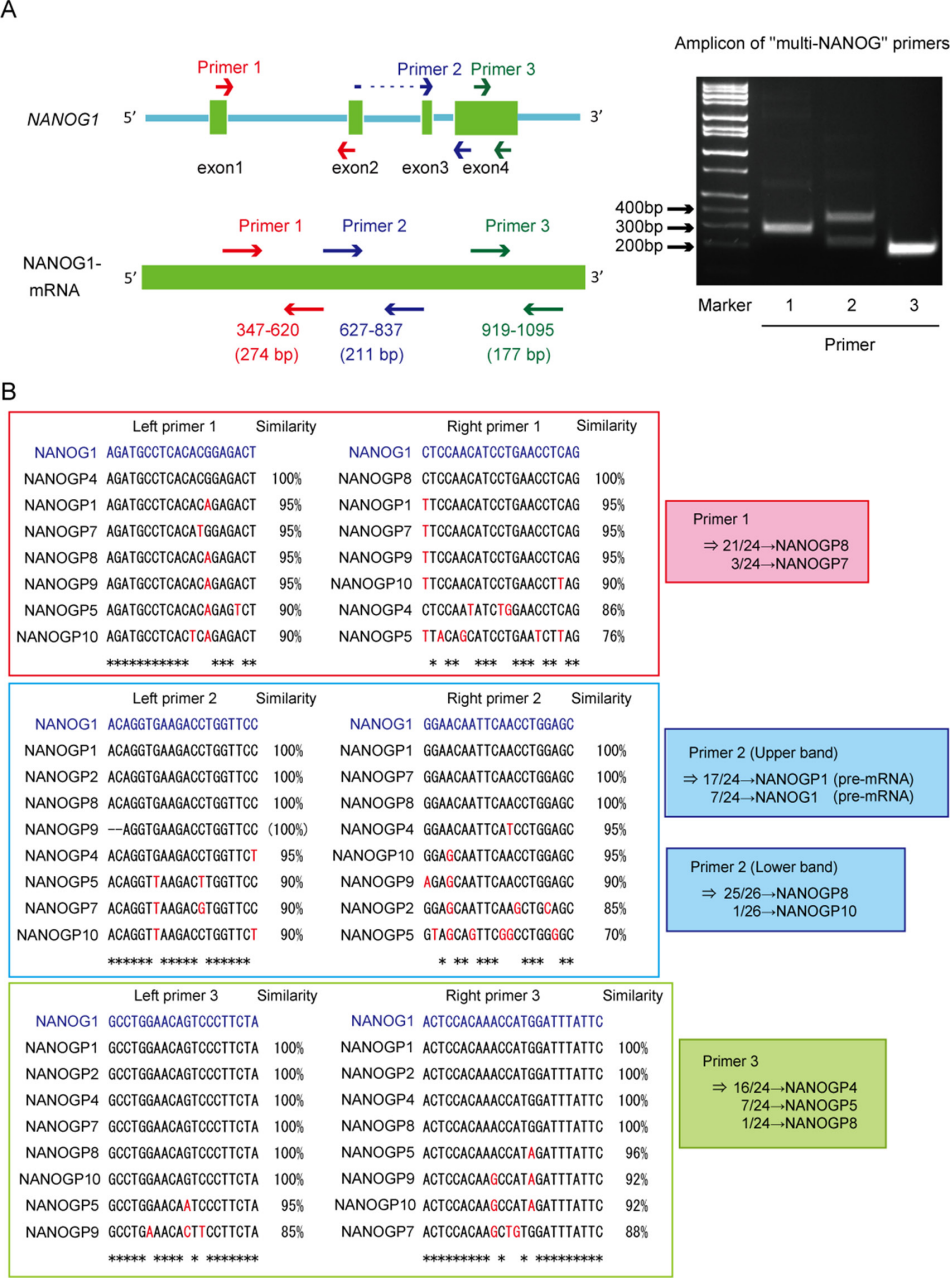
Unequal amplification of transcripts corresponding to *NANOG1* and its pseudogenes by PCR **A.** Left panel: Schematic representation of the *NANOG1* gene (top) and NANOG1 mRNA (bottom). Arrows indicate “multi-NANOG” primer positions. DU145 cDNA was amplified using each “multi-NANOG” primer set. Right panel: Amplicons were separated in the depicted agarose gel. **B.** Left panels: Multiple sequence alignments of *NANOG1* and its pseudogenes. Primer sequences are presented in blue at the top of each panel, and base differences between *NANOG1* and its pseudogenes are indicated in red. PCR products generated using each “multi-NANOG” primer set were cloned into plasmids, and their sequences were analyzed. Right panel: Results of sequence analyses.

We analyzed NANOG protein expression in each cell line using Western blot to examine whether *NANOG1* and *NANOGP8* contribute to the production of NANOG protein in DU145 cells. NANOG protein expression decreased significantly in the *NANOG1*- and *NANOGP8*-knockout cell lines (Figure 1G). *NANOG1* is the primary contributor of NANOG expression in ESCs, but NANOG protein is primarily derived from *NANOGP8* in DU145 cells, as shown by PCR-based analyses [35]. Therefore, we designed three “multi-NANOG” primer sets with high similarity to NANOG pseudogenes, with the exception of *NANOGP3* and *NANOGP6*, which are too different from the other NANOG pseudogenes to be amplified by a common primer set (Figure 2A and 2B). Sequence analyses indicated that primer 1 predominantly amplified *NANOGP8*, primer 2 amplified *NANOGP8, NANOGP1* and *NANOG1*, and primer 3 mainly amplified *NANOGP4* and *NANOGP5*. The upper amplicon of primer 2 was derived from *NANOGP1* and *NANOG1* cDNA, which are derived from each pre-mRNA that included intron 3 (Figure 2A). Therefore, we conclude that each primer exhibited a PCR bias (Figure 2A and 2B), and RT-PCR and sequence analyses of cloned cDNA are not appropriate for examining the proportion of NANOG expression from each gene. DU145 cells actually express all 7 NANOG genes, including *NANOG1* and *NANOGP8*. We established a *NANOG1*-rescued cell line and a *NANOGP8*-rescued cell line from *NANOG1*−/− #1 cells and *NANOGP8*−/− #1 cells, respectively, to avoid non-specific effects of the CRISPR/Cas9 system. NANOG protein expression was recovered in each rescued cell line, which suggests that exogenous NANOG1 protein promotes NANOGP8 expression, and vice versa (Figure 1H). Taken together, our results indicate that DU145 cells express both NANOGP8 and NANOG1.

### *NANOG1* and *NANOGP8* knockout decreases the clonogenic potential of DU145 cells

**Figure 3:**
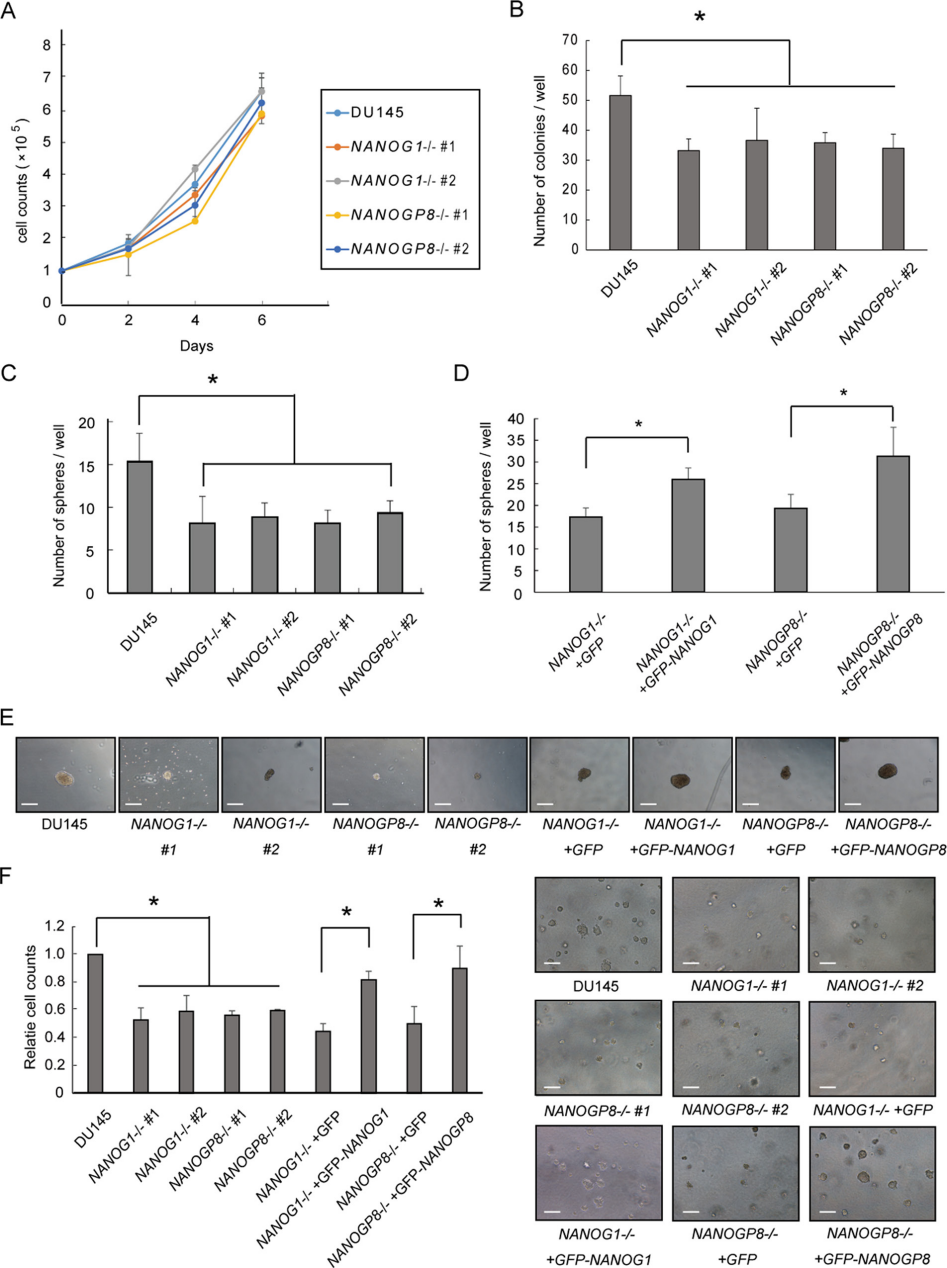
Knockout of *NANOG1* and *NANOGP8* decreases clonogenic potential **A.** Adherent cell growth was assessed by cell counting at the indicated times. Error bars indicate means ± SE (*n* = 3). **B.** Anchorage-dependent colony formation assay. A total of 100 cells were seeded in 6-well plates, and colonies consisting of at least 50 cells were counted after 14 days. The number of colonies for each cell line is compared with the number of colonies for parental DU145 cells. Error bars indicate means ± SE (*n* = 4). *p < 0.05. **C–D.** Sphere-forming assay. Each cell line was plated at 2000 cells/well in low-attachment 6-well plates and cultured in serum-free epithelial basal medium (Cambrex) supplemented with B27, insulin, EGF, and basic fibroblast growth factor for 2 weeks. Sphere-forming capacity was assessed based on the number of spheres observed at 14 days. Error bars indicate means ± SE (*n* = 5). *p < 0.05. C) The numbers of spheres for the *NANOG1*- and *NANOGP8*-knockout cell lines were compared with the number of spheres for DU145 cells. D) The numbers of spheres for the *NANOG1*- and *NANOGP8*-rescued cell lines were compared with the numbers of spheres for the GFP-expressing knockout cell lines. **E.** Representative microscopic images of sphere formation for the indicated cell lines. Scale bars represent 300 μm. **F.** Clonogenic potential under anchorage-independent growth conditions was examined using the soft agar colony formation assay. The microscopic appearance of each cell line in soft agar is depicted on the right side of the figure. Each relative cell count at 8 days was calculated as a ratio compared with DU145 cells. Error bars indicate means ± SE (*n* = 3). *p < 0.05. Scale bars represent 300 μm.

Malignant tumor cells exhibit well-known aggressive and anchorage-independent cell growth, and a high clonogenic potential. We first examined *NANOG1*−/− and *NANOGP8*−/− cell proliferation in a monolayer to examine whether *NANOG1* and *NANOGP8* were involved in these well-known tumor cell properties. However, each knockout cell line exhibited a similar growth rate as DU145 cells (Figure 3A). NANOGP8-expressing prostate cancer cells exhibit greater clonogenic potential [30]. We utilized a colony formation assay to examine the role of *NANOG1* and *NANOGP8* in self-renewal. The colony-forming capacity of NANOG1−/− and NANOGP8−/− cells was decreased compared to parental DU145 cells (Figure 3B). The sphere-forming capacity of prostate cancer cells is highly associated with tumorigenic potential [34]. Therefore, we examined the sphere-forming capacity of *NANOG1*−/− and *NANOGP8*−/− cells and evaluated the number of spheres that formed after 2 weeks. Five independent experiments demonstrated that the sphere-forming capacity of *NANOG1*−/− and *NANOGP8*−/− cells decreased to approximately 50% compared to DU145 cells (Figure 3C). We also evaluated the sphere-forming capacity of *NANOG1*- and *NANOGP8*-rescued cell lines and demonstrated that the number of formed spheres in *NANOG1*- and *NANOGP8*-rescued cell lines increased significantly compared to the non-rescued cell lines (Figure 3D). The size of the formed spheres in *NANOG1*−/− and *NANOGP8*−/− cell lines was generally smaller than the parental DU145 cells, and this phenotype was also recovered in *NANOG1*- and *NANOGP8*-rescued cell lines (Figure 3E). We also utilized the soft agar colony formation assay to examine clonogenic potential under anchorage-independent conditions. The clonogenic potential under anchorage-independent conditions was lower in the *NANOG1*−/− and *NANOGP8*−/− cell lines compared to DU145 cells. This phenotype was also recovered in *NANOG1*- and *NANOGP8*-rescued cell lines (Figure 3F). Therefore, we concluded that *NANOG1* and *NANOGP8* increase the clonogenic potential of DU145 cells.

### *NANOG1* and *NANOGP8* knockout decreases DU145 cell migration capability

**Figure 4:**
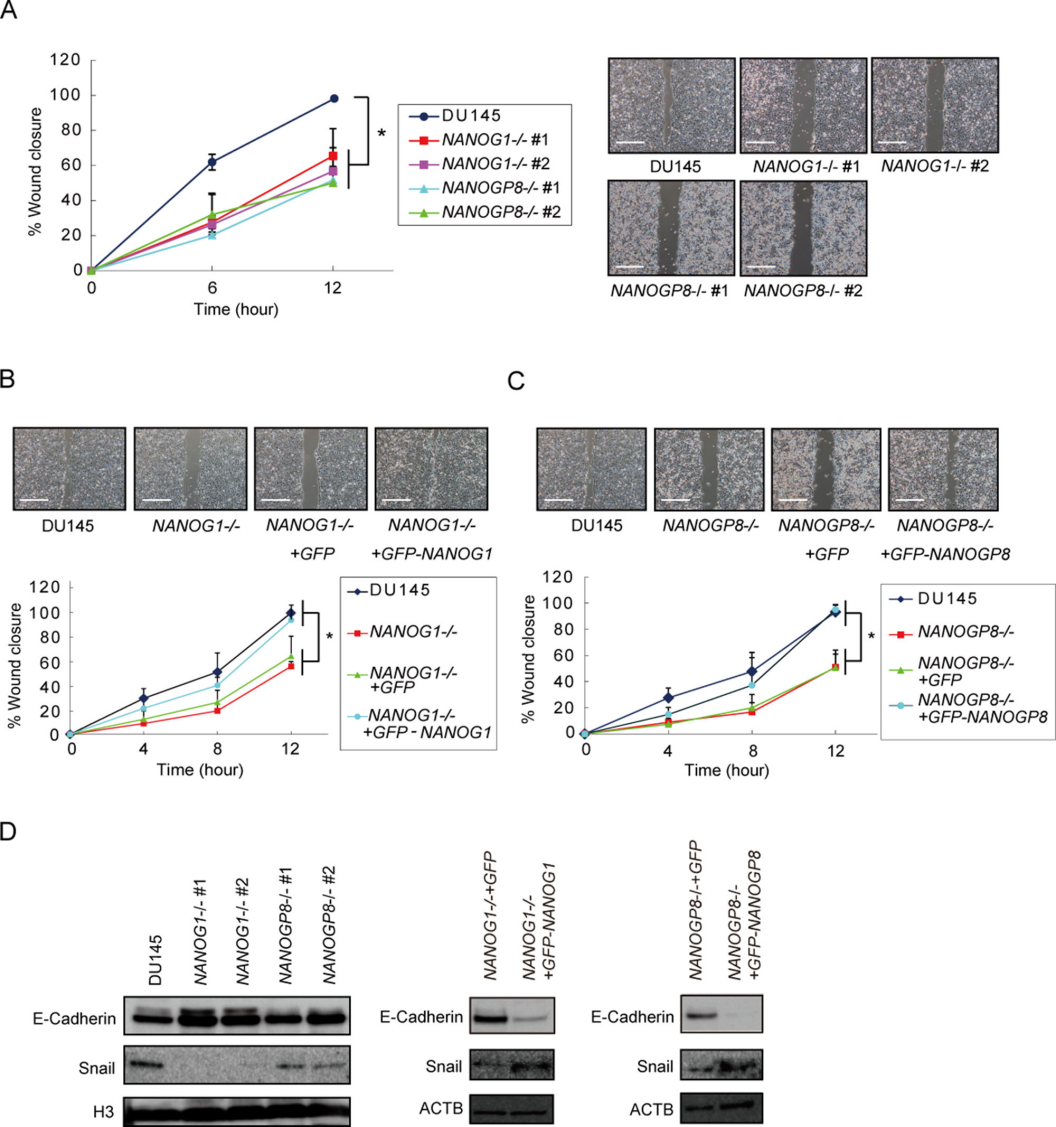
Knockout of *NANOG1* and *NANOGP8* decreases migratory potential **A–C.** Wound healing assay in the indicated cell lines. The width of wound closure in DU145 cells after 12 hours was set to 100%. Error bars indicate means ± SE (*n* = 3). *p < 0.05. Scale bars represent 500 μm. **D.** E-cadherin and Snail expression levels in the indicated cell lines. E-cadherin and Snail expression were analyzed by Western blot. H3 and ACTB were used as loading controls.

NANOG controls cell migration in cancer [19] [38]. Therefore, wound-healing assays were performed to examine the effect of *NANOG1* and *NANOGP8* on prostate cancer cell migration. Migration was decreased in *NANOG1*−/− and *NANOGP8*−/− cells by 40–60%, and this phenotype was recovered in the *NANOG1*- and *NANOGP8*-rescued cell lines. The rescued cell lines exhibited similar migration to parental DU145 cells in this study (Figure 4A–4C). Decreased E-cadherin expression in cancer cells leads to epithelial-mesenchymal transition (EMT) [39]. The transcription factor Snail represses E-cadherin expression [40] and accelerated EMT promotes cell migration. *SNAIL*, which encodes SNAIL, is recently reported to be a transcriptional target of NANOG in reprograming cells [41]. We also found that *NANOG1*−/− and *NANOP8*−/− DU145 cells exhibited increased E-cadherin expression and decreased Snail expression and that the increased E-cadherin and decreased Snail expression were abolished in *NANOG1*- and *NANOGP8*-rescued cells (Figure 4D). These results indicate that *NANOG1* and *NANOGP8* are involved in the promotion of migration capacity in DU145 cells.

### *NANOG1* and *NANOGP8* knockout increases docetaxel sensitivity in DU145 cells

**Figure 5:**
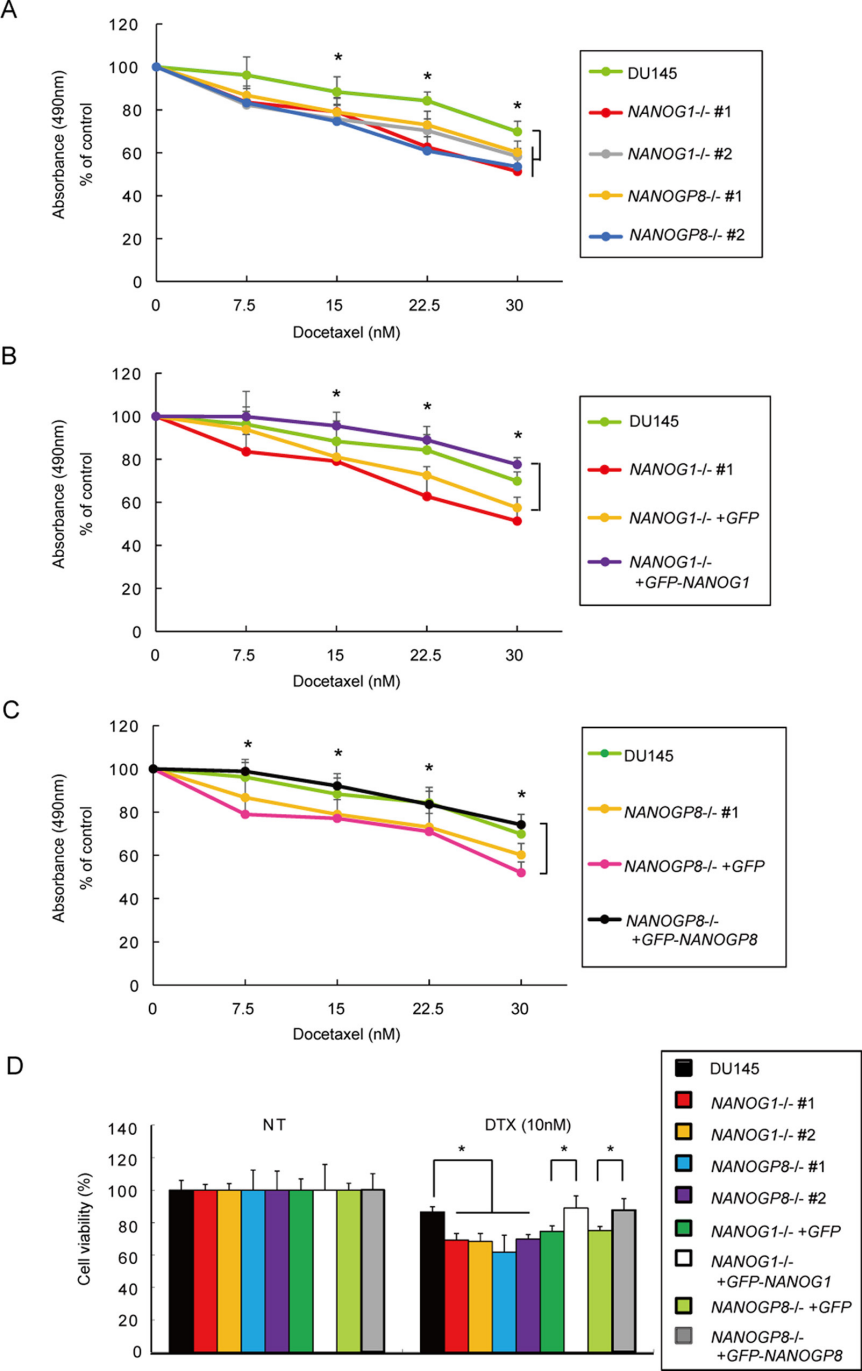
Knockout of *NANOG1* and *NANOGP8* increases sensitivity to docetaxel **A–C.** Cell viability was assessed by an MTS assay after docetaxel administration. Each cell line was seeded in a 48-well plate at 2.5 × 10^4^ cells/well; 24 hours later, docetaxel was added at concentrations of 7.5, 15, 22.5, or 30 nM. Cell viability was evaluated after 48 hours of docetaxel treatment. Error bars indicate means ± SE (*n* = 9), and data are presented as percentages relative to the indicated cell lines. *p < 0.05. **D.** Each cell line was seeded in a 6-well plate at 5 × 10^4^ cells/well; 24 hours later, docetaxel was added at a concentration of 10 nM. Residual cell numbers were counted 72 hours after the administration of 10 nM docetaxel (DTX). NT, no treatment. Error bars indicate means ± SE (*n* = 3), and the data are presented as percentages relative to the indicated cell lines. *p < 0.05.

*NANOG1* and *NANOGP8* overexpression increases drug resistance in cancer cells [30], and NANOG knockdown increases drug sensitivity in cancer cells [42]. Therefore, we evaluated the viability of *NANOG1*−/− and *NANOGP8*−/− cells using an MTS assay 48 hours after docetaxel administration. *NANOG1*- and *NANOP8*-knockout cells showed increased sensitivity to docetaxel (Figure 5A), and docetaxel sensitivity was similar between the *NANOG1*- and *NANOGP8*-rescued cell lines and parental cells (Figure 5B and 5C). We also evaluated the number of residual cells from each cell line 72 hours after 10 nM docetaxel treatment *in vitro* (Figure 5D). These results indicate that *NANOG1* and *NANOGP8* decrease drug sensitivity in DU145 cells.

### *NANOG1* and *NANOGP8* knockout decreases the *in vivo* tumorigenicity of DU145 cells

**Figure 6:**
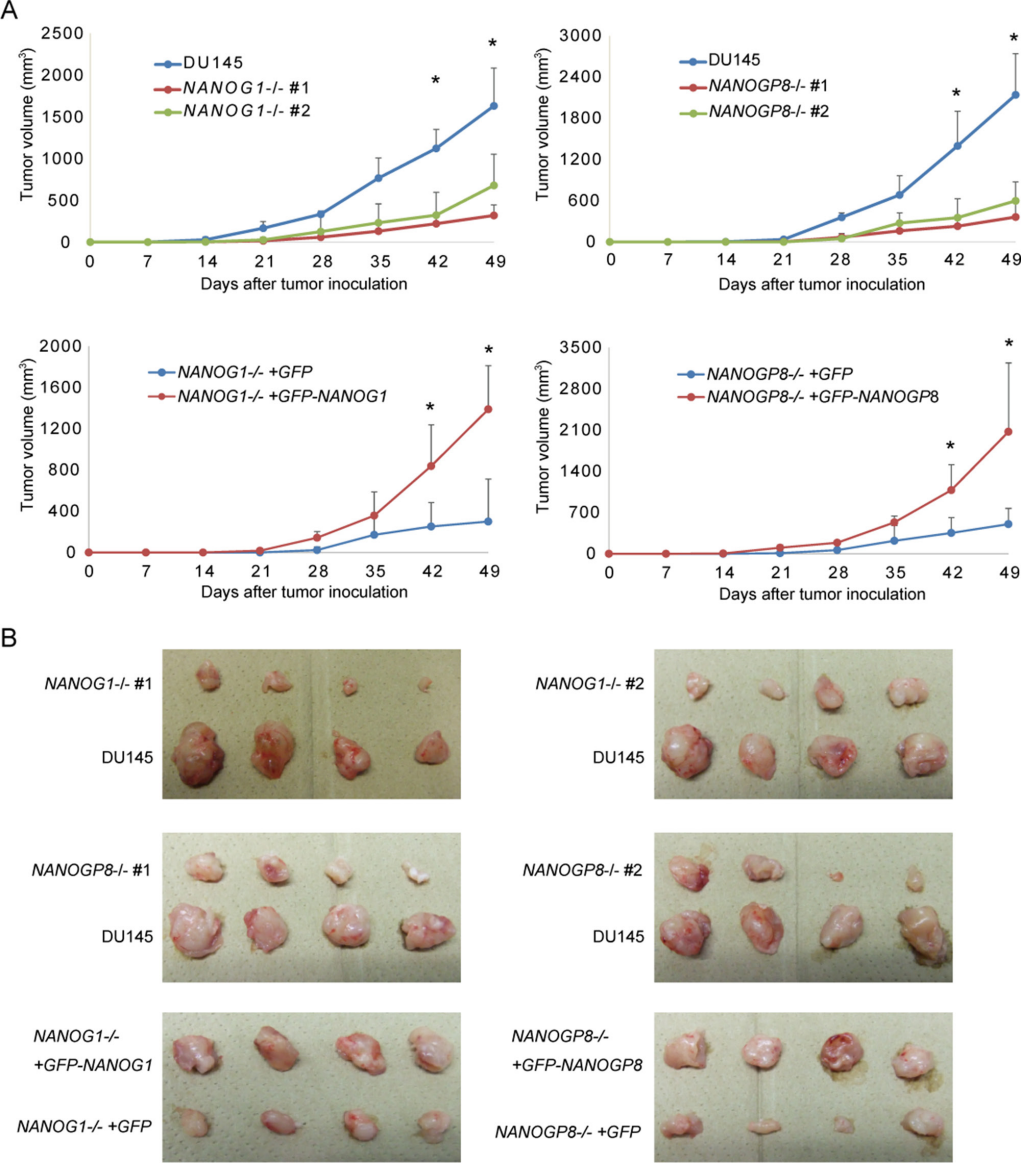
Tumorigenic potential was reduced in *NANOG1*- and *NANOGP8*-knockout DU145 cells **A.** Tumor development *in vivo*. DU145 and its derivatives (2 × 10^6^ cells) were implanted subcutaneously in NOD-SCID mice. Tumor appearance and development were observed for 7 weeks. Tumor volume was calculated using the following formula: tumor volume (mm^3^) = length × (width)^2^ /2. The data for the indicated time points are presented as means ± SE (*n* = 4). *p < 0.05. **B.** Tumor images in the indicated cell lines 49 days after inoculation.

Tumorigenic potential is promoted by *NANOGP8*, but not *NANOG1*, overexpression in cancer cells [30]. We subcutaneously injected 2 × 10^6^ *NANOG1*-knockout cells, *NANOGP8*-knockout cells, or parental cells into non-obese diabetic/severe combined immunodeficient (NOD-SCID) mice to determine whether NANOG depletion influences tumor development *in vivo*. *NANOG1*- and *NANOGP8*-knockout cells exhibited significantly decreased tumorigenic potential compared to parental cells (Figure 6A and 6B). We next examined whether this phenotype was recovered by exogenous *NANOG1* and *NANOGP8* expression. *NANOG1*- and *NANOGP8*-rescued cells showed increased tumorigenic potential compared to *NANOG1*−/−+*GFP* and *NANOGP8*−/−+*GFP* cells, respectively (Figure 6A and 6B). These results demonstrated that *NANOGP8* and *NANOG1* increase the tumorigenic potential of DU145 cells *in vivo*.

## DISCUSSION

Elucidating the link between transcription factors and cancer properties is of interest because improves our understanding of how cancer cells promote malignancy through the regulation of gene expression pattern. NANOG1 is a critical transcription factor that enables the pluripotent properties of ESCs [43] [44]. Recent studies suggested a role for NANOG1 in the progression of malignancy using knockdown systems [16] [19] [42]. *NANOG1* knockdown using RNAi likely exerts an off-target effect on *NANOGP8* mRNA, and vice versa, because of the high similarity between NANOG1 and NANOGP8 mRNA. Moreover, knockdown using RNAi cannot completely turn off target genes, and the possibility that cells with residual gene function influence other cells cannot be excluded, especially in tumor development. Therefore, knockdown experiments are not suitable for functional analyses of *NANOG1* and *NANOGP8* genes. Jeter et al. reported that the overexpression of *NANOGP8*, but not *NANOG1*, in prostate cancer cell lines increases migration capacity and tumorigenic potential [30]. However, Lu et al. reported that *NANOG1* overexpression in breast cancer cells increases migration capacity, metastasis, and tumorigenic potential [27], and Siu et al. reported that *NANOG1* overexpression increased the migration capacity of an ovarian cancer cell line [19]. Therefore, the effects of *NANOG1* and *NANOGP8* on malignant potential remain controversial. This study used the CRISPR/Cas9 system to establish *NANOG1*- and *NANOGP8*-knockout prostate cancer cell lines and examine the function of *NANOG1* and *NANOGP8* genes in prostate cancer cells. We found that DU145 cells express *NANOGP8* and *NANOG1*, in contrast to an earlier report [35]. As mentioned above (Figure 2), we demonstrated that RT-PCR and cloning analyses are not suitable for the quantification of the expression of genes with many pseudogenes with highly similar mRNA sequences, such as *NANOG1*, because primers do not evenly amplify each cDNA despite perfectly matched primer-target sequences. Our results revealed an equivalent proportion of NANOG1 and NANOGP8 protein expression and an equivalent function of *NANOG1* and *NANOGP8* genes in the regulation of the malignant potential of DU145 prostate cancer cells.

Notably, one double-strand break (DSB) caused by one gRNA in *NANOG1*-knockout prostate cancer cells led to the same length deletion in both alleles in the establishment of knockout cell lines using the CRISPR/Cas9 system, and two DSBs caused by two gRNAs in *NANOGP8*-knockout cells led to different length deletions on each allele. In general, a DSB in mammalian cells can be repaired by one of two general repair pathways, non-homologous end-joining (NHEJ) and homologous recombination (HR) [45], and a DSB at a target locus induced by CRISPR/Cas9 can be repaired through either NHEJ or homology-directed repair (HDR) [46]. DSBs caused by CRISPR/Cas9 are repaired through NHEJ in the absence of a homologous repair donor, which results in indels [46]. Both alleles should have different indels because NHEJ of the DSBs in both alleles are independently repaired. Therefore, we speculate that CRISPR/Cas9-mediated DSBs at the same sites in both alleles may be repaired by HR after the repair of one allele by the NHEJ pathway, which results in *NANOG1*-knockout cells with the same deletions on both alleles. The DNA deletion resulting from the two CRISPR/Cas9-mediated DSBs at separate target sites in *NANOGP8*-knockout cells was longer. Therefore, it may be more difficult to repair these DSBs through the HR pathway, which results in different deletions on each allele.

*NANOG1*- and *NANOGP8*-rescued cell lines exhibited increased endogenous NANOG protein expression compared to the parental knockout cell lines (Figure 1H). The increased endogenous NANOG protein in the *NANOG1*-rescued, *NANOG1*-knockout cell line was likely derived from *NANOGP8*, and vice versa, because only *NANOG1* and the NANOG pseudogene *NANOGP8* encode full-length NANOG protein. The NANOG1 protein binds to its own gene promoter and promotes its own expression in ESCs [44]. Therefore, we hypothesize that *NANOG1* and *NANOGP8* reciprocally promote each other's expression in prostate cancer cells. The reciprocal promotion between *NANOG1* and *NANOGP8* may be important for the maintenance of malignant potential. Notably, we could not detect substantial NANOG-GFP expression in either *NANOG1*- or *NANOGP8*-rescued cell lines using fluorescence microscopy, which suggests that excessive NANOG protein expression is lethal to somatic cancer cells, as speculated previously [30].

In our study, knockout of *NANOG1* and *NANOGP8* did not alter cell growth, but knockout of each gene significantly decreased malignant potential, including colony formation (Figure 3B, 3C, and 3F), migration (Figure 4A), drug resistance (Figure 5A), and tumorigenicity (Figure 6A). These results suggest that NANOG activates cancer cell properties but not cell growth. These data are consistent with the function of *NANOG1* in ESCs, in which NANOG1 forms a core module that regulates genes related to cell properties, and cell growth-related genes are regulated by a MYC module [47].

Our results demonstrated that *NANOG1* and *NANOGP8* activate migration capacity via E-cadherin expression. *NANOG1* overexpression in ovarian cancer cells has been shown to enhance migration capacity, which is accompanied by decreased E-cadherin, caveolin-1, FOXJ1, and FOXO1 expression [19]. In addition, TALEN-mediated *NANOG1* deletion in HeLa cells decreases migration capacity, which is accompanied by increased E-cadherin expression and decreased N-cadherin and vimentin expression [48]. Furthermore, E-cadherin promotes epithelial cell-cell adhesions, and decreased E-cadherin expression is important for EMT [39].

Our data indicate that *NANOG1* and *NANOGP8* are relevant to drug resistance. Previous studies demonstrated that *NANOG1* and *NANOGP8* overexpression in MCF7 cells upregulates several detoxification genes, including ABCG2, Bcl-2, ALDH1A1, and CD133 [30], and disruption of *NANOG1* decreases the expression of *MDR1* [48]. *MDR1* encodes a P-glycoprotein that pumps various foreign substances out of cells. Various studies showed that *NANOG1* depletion decreases tumorigenicity [16, 48], and *NANOG1* overexpression increases tumorigenicity [27]. Lu et al. used an inducible *NANOG1* transgenic mouse model and reported that ectopic *NANOG1* expression upregulates the PDGFRa gene [27], which encodes an alpha-type platelet-derived growth factor receptor that drives tumorigenesis and metastasis in various cancers [49]. Emerging evidence also suggests that *NANOGP8* overexpression increases tumorigenicity [30, 33]. Our results indicate that *NANOG1* and *NANOGP8* are involved in the tumorigenic potential of prostate cancer cells, which is consistent with these previous studies.

In our study, the phenotypes (e.g., sphere formation capacity, migration capability, drug resistance, and tumorigenic potential) of *NANOG1*- and *NANOGP8*-rescued cell lines only recovered to the levels of the parental DU145 cell line despite an excess expression of NANOG protein in the rescued cell lines. We hypothesize that this effect resulted from a loss of the reciprocal promotion between *NANOG1* and *NANOGP8* in the *NANOG1*- and *NANOGP8*-rescued cell lines.

We attempted to establish a *NANOG1*- and *NANOGP8*-double knockout cell line to examine whether an *NANOG1* and *NANOGP8* double knockout exerted a positive effect on the loss of malignant potential. However, we were unsuccessful in our attempt. We isolated more than 300 colonies that were candidates for the double knockout, but we did not find cells with deletions of both *NANOG1* and *NANOGP8* on both alleles. We identified 2 *NANOG1*-knockout cells out of 46 candidate colonies and 2 *NANOGP8*-knockout cells out of 24 candidate colonies, which suggests that the knockout of both *NANOG1* and *NANOGP8* genes in DU145 cells is lethal (Supplementary Figure 1).

In summary, we established *NANOG1*- and *NANOGP8*-knockout prostate cancer cell lines using the CRISPR/Cas9 system. Our results indicate that *NANOG1* and *NANOGP8* are expressed equally and that both genes activate many properties that are associated with the malignant potential in prostate cancer cells, including sphere formation capacity, migration capability, drug resistance, and tumorigenic potential.

## MATERIALS AND METHODS

### Cell culture

The androgen-independent human prostate cell line DU145 was purchased from the American Type Culture Collection (Rockville, MD). DU145 and its derivatives were cultured in RPMI-1640 medium (Nacalai Tesque) supplemented with 10% FBS (Biowest), 100 U/ml penicillin, and 100 μg/ml streptomycin (Penicillin-streptomycin mixed solution; Nacalai Tesque). These cells were incubated at 37°C in a humidified atmosphere of 95% air and 5% CO_2_. Growth curves were generated for each cell line as follows. Each cell line was seeded in a 6-well plate at 1 × 10^5^ cells/well and incubated for up to 6 days. Cell growth was assessed by cell counting every other day. Cells were trypsinized before reaching 70% confluency and transferred to 10-cm dishes. All experiments were conducted using passage-matched parental cells.

### Establishment of DU145*^NANOG1−/−^*, DU145*^NANOGP8−/−^*, and NANOG-rescue cell lines

The targeted gRNA expression oligos were introduced into the pX330 vector [46] (Addgene). The sequences of these oligos are shown in Supplementary Table 1. A mixture of 1 μg of pX330 plasmid DNA containing each target gRNA sequence and 0.5 μg of pPGKpuro (Addgene) was transfected into suspended DU145 (1 × 10^5^ cells) [36]. NEON (Invitrogen) electroporation was used to transfect the plasmids, and transfected cells were cultured in medium containing 1.0 μg/ml puromycin for 2 days for selection. Surviving cells were trypsinized and diluted in medium for colony formation. Single colonies were selected, and each colony was passaged and genotyped. DNA was isolated using a DNeasy Blood & Tissue Kit (Qiagen). The genomic region surrounding the CRISPR/Cas9 target site for each gene was PCR amplified, and PCR products were purified using a QIAquick Gel Extraction Kit (QIAGEN) according to the manufacturer's protocol. The amplicons were cloned into the pCR-BluntII-TOPO vector (Invitrogen). Each colony was selected, and the amplicon sequences were analyzed using a 3100 Genetic Analyzer (ABI). Supplementary Table 1 shows the primer sequences.

NANOG-rescued DU145 cell lines and control cell lines were generated by transducing 30 μg of plasmid (CAG-GFP-*NANOG1*-IRES-Puro, CAG-GFP-*NANOGP8*-IRES-Puro, and -*GFP*-IRES-Puro) into suspended *NANOG1*−/− cells and *NANOGP8*−/− cells (2 × 10^7^ cells). Single colonies were selected after a 0.5-μg/ml puromycin selection. NANOG protein expression levels in each cell line were examined using Western blotting.

### *In vivo* tumorigenicity experiments

DU145 and its derivatives (2 × 10^6^ cells) were implanted subcutaneously in NOD-SCID mice. These NOD-SCID mice, aged 5 weeks, were purchased from CLEA Japan and maintained in a temperature-controlled, pathogen-free room. All animals were handled according to approved protocols and the guidelines of the Animal Committee of Osaka University (Osaka, Japan). Mouse appearance and tumor development were observed for 7 weeks. Tumor volume was calculated according to the following formula: tumor volume (mm^3^) = length × (width)^2^/2 [50].

### Soft agar colony formation assay

A CytoSelect 96-well Cell Transformation Assay (Soft Agar Colony Formation) Kit (Cell Biolabs) was used to evaluate anchorage-independent growth according to the manufacturer's instructions. Briefly, DU145 and its derivatives (1.5 × 10^3^ cells) were mixed with an agar solution, seeded into wells, and culture medium was added to each well. Cells were incubated at 37°C in a humidified atmosphere of 95% air and 5% CO_2_ for 8 days. The colonies were lysed after agar solubilization, and CyQuant GR Dye was used to quantify anchorage-independent growth using a 485/520 nm filter set.

### Sphere culture

Spheres of DU145 and its derivatives formed as previously described [51]. Briefly, each cell line (2 × 10^3^ cells) was plated on low-attachment 6-well dishes (Corning). Cells were cultured in a serum-free epithelial basal medium (Cambrex) supplemented with B27, 4 μg/ml insulin (Sigma-Aldrich), 20 ng/ml EGF, and 20 ng/ml basic fibroblast growth factor (bFGF; Invitrogen) for 2 weeks. Sphere forming capacity was assessed based on the number of colonies.

### MTS assay

Docetaxel was purchased from Aventis Pharmaceuticals (Sanofi-Aventis). Each cell line was seeded in a 48-well plate at 2.5 × 10^4^ cells/well, and docetaxel was added at concentrations of 7.5, 15, 22.5, or 30 nM 24 hours later. Cell viability was evaluated after 48 hours of docetaxel treatment using a CellTiter 96 Aqueous One Solution Cell Proliferation Assay Kit (Promega) as previously described [52].

### Migration assay

Migration ability was measured using a Culture-Insert in a μ-Dish 35 mm (Ibidi) as previously described [53]. Briefly, each cell line was suspended at a concentration of 5 × 10^5^ cells/μl, and 3.5 × 10^4^ cells were placed in each well. The wells were removed gently after a 24-hour incubation. The width of the scratch was measured at the beginning and every 4–6 hours during cell migration, and the wound closure rate was quantified as previously described [54].

### Western blot analysis

Anti-human NANOG (D73G4), anti-human ACTB (13E5), anti-human H3 (3H1), and anti-human Snail (C15D3) antibodies were purchased from Cell Signaling Technology. The anti-human E-cadherin (CD324) antibody was purchased from BD Biosciences. Cell lysates were separated using SDS-PAGE, and separated proteins were transferred onto polyvinylidene difluoride (PVDF) membranes. The membranes were blocked with 3% skim milk and incubated overnight at 4°C with the primary antibodies. Signals were detected using Chemi-Lumi One or Chemi-Lumi One Super (Nacalai Tesque) and an ImageQuant LAS 4000 mini system (GE Healthcare).

### Statistical analysis

The results are reported as the mean ± SD. A two-tailed unpaired Student's *t*-test was used to determine the statistical significance of differences between two groups, and Tukey's test was used to determine the statistical significance of differences between more than three groups. Probability values of *p* < 0.05 were considered statistically significant. Statistical analyses were conducted using JMP9 (SAS Institute).

## SUPPLEMENTARY FIGURE AND TABLE


