# Supplementary material for: CRISPR/Cas9-mediated gene knockout of NANOG and NANOGP8 decreases the malignant potential of prostate cancer cells
Source: Oncotarget. 2015 Jun 15;6(26):22361–74. doi: 10.18632/oncotarget.4293 (PMC4673169; doi:10.18632/oncotarget.4293)
Supplement: Supplementary file 1 [file oncotarget-06-22361-s001.pdf]

SUPPLEMENTARY FIGURE AND TABLE

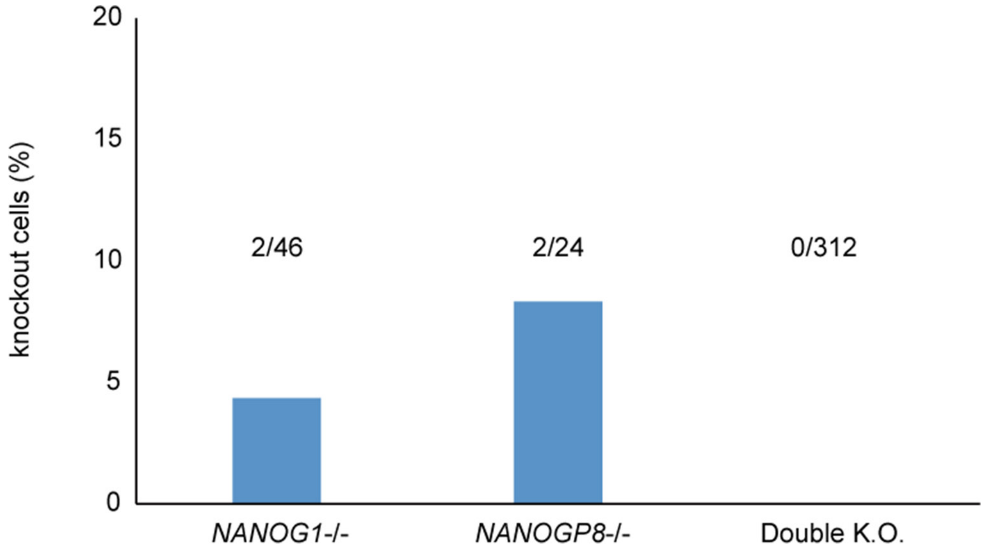

Supplementary Figure 1: The proportion of the knochout cells among candidate cells. We found 2 *NANOG1*-knochout cells out of 46 colonies of candidate, and found 2 *NANOGP8*-knockout cells out 24 colonies of candidate. However, we could not find *NANOG1*- and *NANOGP8*-double knockout cells, instead of picking up more then 300 colonies of candidate.

Supplementary Table 1: Primers used in this research

***NANOG1*_gw_F**	ATCTGGGGTTCTGGGAATTATC	Cloning for *NANOG1* intron1-exon2
*NANOG1*_gw_R	GGTCTGTGCAAGAAAGTGGTTA	Cloning for *NANOG1* intron1-exon2
*NANOGP8*_gw_F1	TGTGTGAAAGTGAGATGGGAAC	Cloning for sequence analysis of *NANOGP8* gene knockout
*NANOGP8*_gw_R1	ACACTCGGTGAAATCAGGGTAA	Cloning for sequence analysis of *NANOGP8* gene knockout
*NANOGP8*_gw_F2	CTTGCTTTGAAGAATCCGACTG	Cloning for sequence analysis of *NANOGP8* gene knockout
*NANOGP8*_gw_R2	CACAAATCACAGGCATAGGTGA	Cloning for sequence analysis of *NANOGP8* gene knockout
*NANOGP1*_gw_F	GAATCTCTTGAACCTGGGAAGC	Cloning for *NANOGP1* genomic region
*NANOGP1*_gw_R	TGGAGGCTGAGGTATTTCTGTC	Cloning for *NANOGP1* genomic region
*NANOGP2*_gw_F	GCCTGGCTTCAAAGCATCT	Cloning for *NANOGP2* genomic region
*NANOGP2*_gw_R	TCTTGCATCTGTTGAAGGCTGA	Cloning for *NANOGP2* genomic region
*NANOGP3*_gw_F	AGTGCCACCATACATGGCTAAT	Cloning for *NANOGP3* genomic region
*NANOGP3*_gw_R	ACACCATTGCTACCCTTTGG	Cloning for *NANOGP3* genomic region
*NANOGP4*_gw_F	AGGCTTGGCATCATTTCATC	Cloning for *NANOGP4* genomic region
*NANOGP4*_gw_R	ACACAGCTGGGTGGAAGAAAAC	Cloning for *NANOGP4* genomic region
*NANOGP5*_gw_F	CATCCAGCTTGTCCCAAAACC	Cloning for *NANOGP5* genomic region
*NANOGP5*_gw_R	TGGAGGCTGAGGTATTTCTGTC	Cloning for *NANOGP5* genomic region
*NANOGP6*_gw_F	CCAAAGCCTGCCTTATTCTAAA	Cloning for *NANOGP6* genomic region
*NANOGP6*_gw_R	GGGTATTGGAAAGTTCTTGCAG	Cloning for *NANOGP6* genomic region
*NANOGP7*_gw_F	CCAACGCATCCGTCTGTAA	Cloning for *NANOGP7* genomic region
*NANOGP7*_gw_R	CATCTGCTGGAGGCTGAAGTAT	Cloning for *NANOGP7* genomic region
*NANOGP8*_gw_F	CTTGCTTTGAAGAATCCGACTG	Cloning for *NANOGP8* genomic region
*NANOGP8*_gw_R	TGGAGGCTGAGGTATTTCTGTC	Cloning for *NANOGP8* genomic region
*NANOGP9*_gw_F	AAGCTGTTTTGCTAGACTGAGCTG	Cloning for *NANOGP9* genomic region
*NANOGP9*_gw_R	TCTGGTCTTCTGTTTCTTGACTGG	Cloning for *NANOGP9* genomic region
*NANOGP10*_gw_F	GGGATGGAATGAGGAATGTG	Cloning for *NANOGP10* genomic region
*NANOGP10*_gw_R	GTGGAACGGAACACAGTTTGG	Cloning for *NANOGP10* genomic region
multi-NANOG_F1	AGATGCCTCACACGGAGACT	Cloning for *NANOG1*' and its pseudogene' transcripts
multi-NANOG_R1	CTCCAACATCCTGAACCTCAG	Cloning for *NANOG1*' and its pseudogene' transcripts
multi-NANOG_F2	ACAGGTGAAGACCTGGTTCC	Cloning for *NANOG1*' and its pseudogene' transcripts
multi-NANOG_R2	GGAACAATTCAACCTGGAGC	Cloning for *NANOG1*' and its pseudogene' transcripts
multi-NANOG_F3	GCCTGGAACAGTCCCTTCTA	Cloning for *NANOG1*' and its pseudogene' transcripts
multi-NANOG_R3	ACTCCACAAACCATGGATTTATTC	Cloning for *NANOG1*' and its pseudogene' transcripts
